# Navigating the Economic Burden of Multiple Myeloma: Insights into Cost-effectiveness of CAR-T and Bispecific Antibody Therapies

**DOI:** 10.1007/s11899-024-00748-5

**Published:** 2025-01-04

**Authors:** Praneeth Reddy Keesari, Diana Samuels, Charan Thej Reddy Vegivinti, Yashwitha Sai Pulakurthi, Revathi Kudithi, Meekoo Dhar, Murali Janakiram

**Affiliations:** 1 Department of Internal Medicine, Staten Island University Hospital, Staten Island, NY USA; 2 Department of Pharmacy, City of Hope National Medical Center, Duarte, CA USA; 3 Department of Hematology-Oncology, The University of Texas MD Anderson Cancer Center, Houston, TX USA; 4 Department of Internal Medicine, New York Medical College - Saint Michael’s Medical Center, Newark, NJ USA; 5 Department of Internal Medicine, Kakatiya Medical College, Warangal, Telangana India; 6 Department of Hematology-Oncology, Staten Island University Hospital, Staten Island, NY USA; 7 Division of Myeloma, Department of Hematology & Hematopoietic Cell Transplantation, City of Hope National Medical Center, 1500 East Duarte Road, Duarte, CA 91010 USA

**Keywords:** Multiple Myeloma, Economic, Cost-effectiveness, CAR-T, Bispecific antibodies

## Abstract

**Purpose of Review:**

Multiple myeloma is a chronic malignancy and with evolving treatment options, understanding the economic burden and cost-effectiveness of therapies is crucial for clinicians and researchers.

**Recent Findings:**

In this, we review the recent approval of Bispecific antibodies and CAR-T for myeloma and their cost implications, including direct and indirect costs. We compare this to current regimens and provide cost comparisons in this review.

**Summary:**

We conclude that the use of more effective therapies such as CAR-T in earlier lines of therapies may be more cost-effective depending on the country and model used. Further studies are essential to better understand the cost-effectiveness of bispecific antibodies including head-to-head comparisons to CAR-T therapy.

## Introduction

Multiple myeloma is a hematologic malignancy with clonal proliferation of plasma cells in bone marrow leading to monoclonal immunoglobulin overproduction and end organ damage. The incidence of multiple myeloma and associated mortality is growing in the U.S with an average lifetime risk of acquiring the disease being around 1 in 100 and overall survival rate of 57% [1]. The average years of life lost to multiple myeloma ranges from 30 years in younger individuals to an overall average of 16.8 years [2]. Multiple myeloma is not only associated with high morbidity and mortality but also imposes a significant economic burden on patients. Prior studies analyzing the economic burden in multiple myeloma patients reported a total cost utilization ranging from 20 to 40 thousand US dollars per patient per month (PPPM) with around 50%—85% being related to multiple myeloma and even higher costs in those exposed to multiple classes of drugs [3–6]. The monthly costs incurred also showed rising trends over the years in these studies. Given significant restrictions and treatment related adverse effects of bone marrow transplantation, multiple newer treatments like immunotherapies have emerged for use in multiple myeloma patients, especially those that have been exposed to multiple (≥ 4) prior classes of drugs. Chimeric antigen receptor T cell (CAR-T) therapy involves genetically modifying patients' T cells to recognize tumor antigens. Bispecific antibodies are drugs that bind to two different antigen sites simultaneously, typically CD3 of T cells and tumor antigens (BCMA/FCRh5/GPRC5D) of the myeloma cells enhancing anti-tumor activity of host immune cells. Since its approval for use CAR-T (2021) and bispecific antibodies (2022) have significantly improved survival in triple class refractory and penta-class refractory multiple myeloma patients [7, 8]. Given heightened healthcare costs, it is important to understand the economic burden and cost-effectiveness of newer therapeutic agents in multiple myeloma patients. In this review we have focused on the economic aspects of use of CAR-T therapy and bispecific antibodies in multiple myeloma patients.

## Economic Burden of Multiple Myeloma

### Direct Costs

Direct costs associated with multiple myeloma treatment are expenses related to drugs, hospitalizations, physician visits, lab work, imaging studies, and other supportive care.

### Drug Cost

The cost of most drugs used in multiple myeloma is approximately 100,000 dollars per year and combination regimens with a Proteosome inhibitor, immunomodulatory agent, and an anti CD-38 targeted monoclonal antibody costs 300,000—600,000 US dollars per year [9]. This cost is expected to rise with disease progression and exposure to multiple classes of drugs. Yang et al. in a retrospective cohort study of 1,521 patients with multiple myeloma of which 66.8% were double class exposed (DCE) and 33.2% were Triple class exposed (TCE) identified that the overall mean total all-cause health care costs were $20,338 PPPM (85% MM-related) of which drug cost accounted to $11,435 PPPM. The drug cost in DCE vs TCE was $9,854 vs $16,117 PPPM and drug cost was the overall cost driver in both populations [3]. In an analysis by Hollmann et al. evaluating the cost burden of relapsed or refractory MM patients treated with triplet regimens over 1 year suggested that the average monthly cost per patient per triplet regimen ranged from $13,784 to $30,657. The study also reported that drug acquisition and treatment duration accounted for the cost of treatment [4]. A study based on 600 multiple myeloma patients receiving treatment in Singapore found that daratumumab based regimens were more expensive than bortezomib based regimens due to unit cost difference but after comparing treatment costs for post progression treatment regimens, patients incurred higher costs following first line bortezomib based regimen than daratumumab [10]. Use of the most effective regimen early in the disease course and use of maintenance therapy after first line treatment to delay progression although may incur higher drug costs but have been found to lower overall costs during the disease course [11–13]. Prior studies have also reported that transitioning treatment in multiple myeloma patients to home-based setting for drug infusions is cost-effective and helps patients spend more time with family [14, 15]. In Table 1 we summarize the drug costs of different lines of therapy for patients with Multiple Myeloma.

**Table 1:** Drug costs of different lines of therapy for Non-Transplant Eligible Multiple Myeloma Patients

Line of Therapy	Treatment regimen ^a^	Approximate Annual Drug Cost for the regimen (USD)^b, c^
**Chemotherapy regimens**		
1st Line	VRd	Bortezomib—$2,159 Lenalidomide—$239,369 Dexamethasone—$574 Total—$242,102
1st Line	DRd	Daratumumab—$165,227 Lenalidomide—$239,369 Dexamethasone—$574 Total—$405,170
1st Line	KRd	Carfilzomib—$173,090 Lenalidomide—$239,369 Dexamethasone—$574 Total—$413,033
1st Line	VCd	Bortezomib—$2,159 Cyclophosphamide—8,087 Dexamethasone—$574 Totak—$10,820
1st Line	D-VRd	Daratumumab—$165,227 Bortezomib—$1,871 Lenalidomide—$239,369 Dexamethasone—$575 Total—$407,042
1st Line	D-KRd	Daratumumab—$165,227 Carfilzomib—$173,090 Lenalidomide—$239,369 Dexamethasone—$575 Total—$578,261
2nd Line	KCd	Carfilzomib—$173,090 Cyclophosphamide—$8,087 Dexamethasone—$574 Total—$181,752
2nd Line	DVd	Daratumumab—$165,227 Bortezomib—$2,158 Dexamethasone—$574 Total—$167,960
2nd Line	SVd	Selinexor—$441,648.00 Bortezomib—$2,158 Dexamethasone—$574 Total—$444,381
**CAR-T**		
2nd Line	Cilta-Cel	$507,687
3rd Line	Ide-Cel	$503,455
**T-cell engagers**		
5th line	Belantamab	$371,658
5th line	Elranatamab	$486,506
5th line	Teclistamab	$234,963
5th line	Talquetamab	$372,380

a—VRd: Bortezomib, Lenalidomide, and Dexamethasone; DRd: Daratumumab, Lenalidomide, and Dexamethasone; KRd: Carfilzomib, Lenalidomide, and Dexamethasone; DVd: Daratumumab, Bortezomib, and Dexamethasone; SVd: Selinexor, Bortezomib, and Dexamethasone; VCd: Bortezomib, Cyclophosphamide, and Dexamethasone; D-VRd: Daratumumab, Bortezomib, Lenalidomide, and Dexamethasone; D-KRd: Daratumumab, Carfilzomib, Lenalidomide, and Dexamethasone; Melphalan flufenamide: Melphalan flufenamide and Dexamethasone; Ide-cel: Idecabtagene vicleucel; Cilta-Cel: Ciltacabtagene autoleucel; Belantamab: Belantamab mafodotin; Selinexor: Selinexor and Dexamethasone; b—Costs calculated using CMS payment limits (Average Sale Price (ASP) plus 6% or wholesale acquisition cost plus 3% when ASP is not yet established) for parenteral drugs and AWP (average wholesale price) for oral medications; Usual recommended dosing without accounting for dose reductions and standard 70 kg, 1.73 m2 body surface area were used for the cost calculations c—https://www.cms.gov/medicare/payment/part-b-drugs/asp-pricing-files; https://www.micromedexsolutions.com/micromedex2/librarian/CS/E7AABD/ND_PR/evidencexpert/ND_P/evidencexpert/DUPLICATIONSHIELDSYNC/ED8554/ND_PG/evidencexpert/ND_B/evidencexpert/ND_AppProduct/evidencexpert/ND_T/evidencexpert/PFActionId/redbook.FindRedBook?navitem=topRedBook&isToolPage=true;

### Healthcare Utilization

Yang et al. reported overall MM-related emergency department visits, outpatient physician office visits, other outpatient visits, visit for outpatient prescriptions, and hospitalizations PPPM were 0.05, 3.22, 4.19, 0.89, and 0.10 respectively with median length of hospital stay being 1.02 days PPPM [3]. The study also reported that the proportion of patients with at least 1 MM-related ED, outpatient, and inpatient visit during the study period were 22%, 55%, and 99% respectively and that healthcare resource utilization was higher in TCE than DCE population even though TCE population had shorter follow-up period. Gupta et al. queried a commercial insurance database between 2016 to 2021 to identify 1492 patients with multiple myeloma on therapy. They had reported that although mean monthly outpatient and inpatient visits (5.0–5.6 OP visits; 0.21–0.32 IP visits) remained stable across advancing Lines of therapy(LOT), the mean all cause monthly costs (not including medication cost) were 16%—35% lower in LOT 1 ($23.6 K) compared to LOT 4($31.8 K) [16]. This may suggest that choosing the most effective regimen for the first LOT may be a cost-effective strategy and reduce the overall treatment cost by lowering inpatient and outpatient visits.

### Indirect Costs

There is limited data on the indirect costs incurred by multiple myeloma patients. Most studies focus on drug costs, inpatient, and outpatient costs when reporting cost burden in multiple myeloma patients. Indirect costs like loss of income, supportive care service costs, disability claims, absenteeism, presenteeism, caregiver absenteeism, loss from early retirement and premature death etc. are often ignored underestimating the economic burden on multiple myeloma patients and the society. While direct medical costs may be easier to obtain from billing and administrative databases, estimating indirect costs involves longitudinal assessment of work-related limitations and productivity losses using standardized questionnaires and tools along with patient reported outcomes. The heterogeneity in economic burden based on patient, disease, treatment, and socioeconomic factors further complicates assessment of indirect costs.

Drug administration time needs to be considered while assessing the economic burden of treatments in multiple myeloma. Traditional chemotherapy regimens often demand significant time commitment from patients, caregivers, and healthcare workers taking up to 65 min for each administration session which is around 24—36 h per year (Table 2). Newer therapies like T-cell engagers would consume around 5 min per session for their administration and around 3 h per year, resulting in a substantial reduction in healthcare resource utilization and potential cost savings (Table 2).

**Table 2:** Administration Time Comparison Between Commonly used Regimens and T-cell Engagers in Multiple Myeloma

Regimen	Drug 1 (Administration time per session)	Drug 2 (Administration time per session)	Drug 3 (Administration time per session)	Total Estimated Administration Time Per Session (Minutes)	Total Estimated Annual Administration Time Per Patient (Hours)
DKD	Daratumumab Subcutaneous (3–5 min)	Carfilzomib (30 min)	Dexamethasone with premeds (30 min)	65	24
KPD	Carfilzomib 30 min	Pomalyst (0 Minutes)	Dexamethasone with premeds (30 min)	60	36
Teclistamab*	Teclistmab (5 min)	N/A	N/A	5	3
Elrantamab*	Elrantamab (5 min)	N/A	N/A	5	3

* = No premedications required; assumes patients have completed step-up dosing; Administration times are estimates based on standard treatment protocols and may vary based on individual patient responses and clinical practice settings

Robinson et al. in an analysis based on ‘a” multicenter randomized control study of 263 relapsed refractory multiple myeloma (RRMM) patients reported that only 10.8% of patients aged ≤ 65 years were working and 37% of those working reported absenteeism of ≥ 1 day over last 4 weeks. The study also noted that 48% of the patients not working attributed it to their RRMM [17]. Merola et al. analyzed around 300 newly diagnosed multiple myeloma patients on oral vs injectable chemotherapy regimens and reported that patients on oral regimens had fewer days of absenteeism (83 vs 110 days) and lower costs incurred from productivity loss ($14,429 vs $18,315) over 1 year period [18]. Another European based cross-sectional study of 115 newly diagnosed multiple myeloma (NDMM) patients undergoing ASCT suggested of 76.5% of the total cohort that were productive before ASCT, only 39.1% were able to maintain productive life post ASCT and average per patient productivity loss over 20-year horizon to be around 290,601 euros [19]. In an analysis on multiple myeloma patients from Portugal, Miguel et al. suggested that indirect costs account to 18% of total cost burden with early retirement being the major driver of this cost and caregiver’s work absence being the least affecting factor [20].

Hence therapies which can 1. Minimize indirect costs to patients 2. Which will continue leading a productive life as desired by patients should also be taken account of in cost analysis.

## Methodologies for Conducting Cost-Effectiveness Analyses in Healthcare

As discussed, the morbidity and mortality of multiple myeloma patients has substantially increased with newer therapies. The cost burden is also significantly raised with these novel therapies which can be related to direct costs, indirect costs, or treatment related adverse effects management costs. Cost-effectiveness analyses are essential for policy makers and clinicians to make informed decisions on the use of these novel therapies like CAR-T therapy and bispecific antibodies for multiple myeloma patients. Cost-effectiveness analysis looks to see if the treatment being analyzed offers health gains relative to its cost. In cost–benefit analysis both costs incurred, and benefits gained are measured in monetary terms for direct comparison. Most cost-effectiveness studies calculate the incremental cost-effectiveness ratio (ICER) which measures the cost per additional year of life gained with the treatment. This is then compared against the willingness to pay (WTP) threshold for the country to determine cost effectiveness of the treatment. Alternatively, studies may report incremental net benefit (INB) which incorporates the willingness to pay and thus a positive INB is considered cost-effective, which is the difference between calculated cost patients are willing to pay for the additional health gain and the actual additional costs with the treatment for the health gains. Multiple pharmacoeconomic models have been used for cost-effective analysis including the Markov model, decision trees, and Monte Carlo model. When estimating cost-effectiveness in patients with multiple myeloma, the Markov model helps researchers estimate the outcomes in terms of how different patients may transition to remission, progression, hospitalization, needing the next line of treatment etc. and hence calculate the associated costs. Decision tree model is a visual representation of outcome probabilities based on treatment options selected after considering the efficacy and adverse effects. As compared to Markov model, it may be simpler to interpret and understand but is typically a short-term analysis and does not include the comprehensive and dynamic effects of treatment on disease state. Monte Carlo method is a more robust computational model that incorporates parameters like response rates, adverse effects, and healthcare costs to run multiple random samples with distribution of these parameters and hence generate the likelihood of outcomes to further estimate the probability of the treatment being cost effective as compared to willingness to pay thresholds. Monte Carlo method incorporates and handles uncertainty by randomly selecting values from probability distribution of the input parameters. It can also predict long term outcomes over multiple time periods like the Markov model [21–24].

## Review of Published Cost-Effectiveness Studies Evaluating CAR-T in Multiple Myeloma

Yamamoto et al. used the Markov and Monte Carlo analysis model on data from LocoMMotion, KarMMa, and CARTITUDE-1 trials to compare the cost-effectiveness of anti BCMA CAR-T [idecabtagene vicleucel(ide-cel) or ciltacabtagene autoleucel(cilta-cel)] for triple class exposed RRMM patients vs no CAR-T (multiagent chemotherapy) strategy over 5 and 10 year timeline in patients in Japan and USA. The analysis included various uncertainties regarding long-term PFS with CAR-T therapy. The study suggested that over a 10-year time horizon CAR-T therapy with cilta-cel may be cost-effective in the USA under a modest PFS of 30% but not ide-cel. The study also noted that BCMA CAR-T therapy was not found to be cost-effective even at the most optimistic PFS over a shorter, 3-year time horizon [25]. Another system-based study by Wu et al. using the Monte Carlo method analyzed the same trials as Yamamoto et al. but MAMMOTH trial instead of LocoMMotion, reported the probability of being cost-effective as 0% and 72% respectively for ide-cel and cilta-cel therapies based on the WTP thresholds established in China [26].

**Fig. 1:**
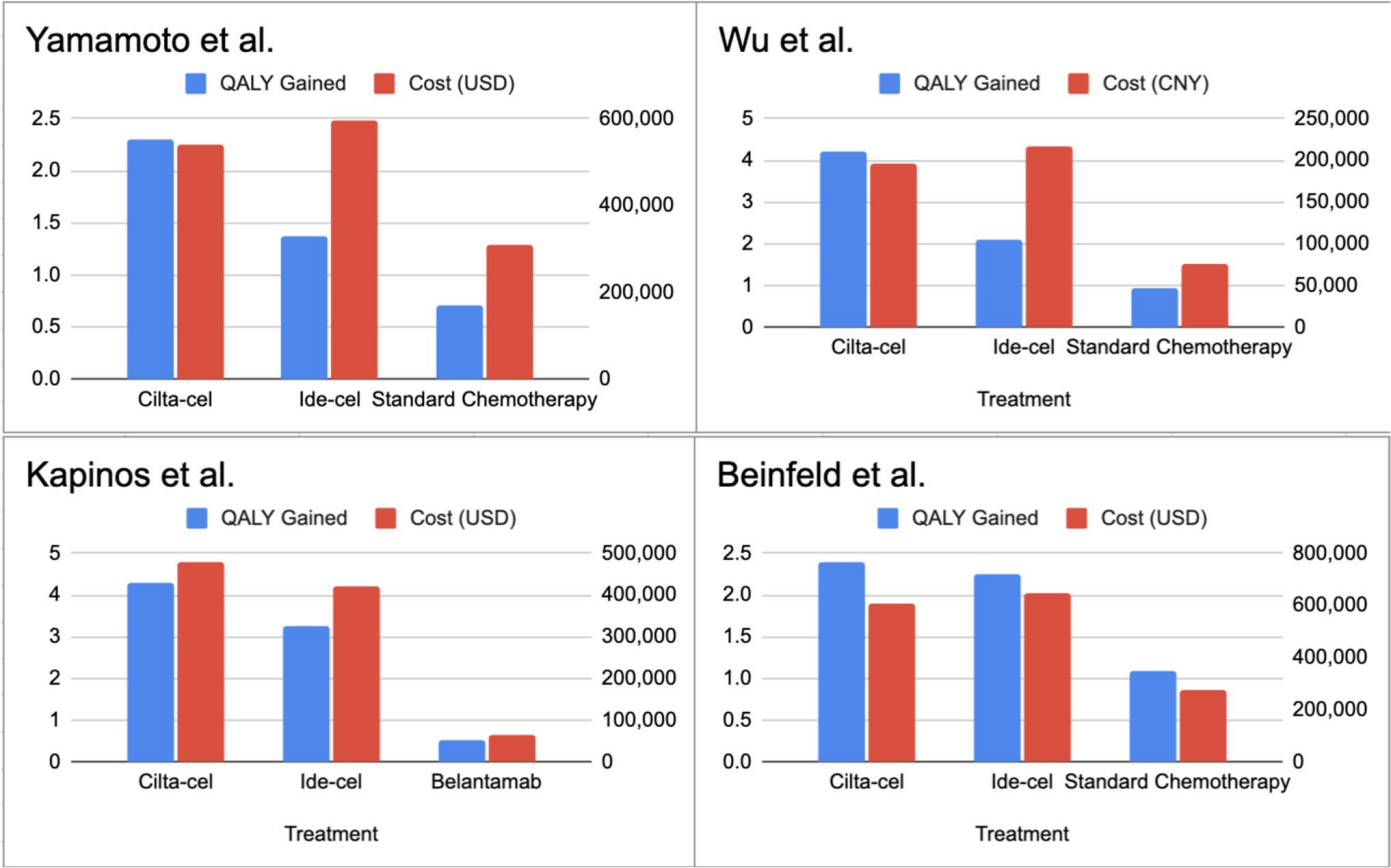
Cost and Quality-Adjusted Life Years (QALY) Gained for Various Treatments in Multiple Myeloma: Comparisons Across Studies. **Cilta-cel:** Ciltacabtagene autoleucel, **Ide-cel:** Idecabtagene vicleucel, **Standard Chemotherapy:** Standard chemotherapy regimens as defined in the respective studies, **Belantamab:** Belantamab mafodotin, QALY gained: Quality adjusted life years gained

Kapinos et al. analyzed data from DREAMM 2, KarMMa, and CARTITUDE-1 trial using Monte Carlo Markov Chain microsimulation model and estimated that CAR-T therapy in triple refractory MM patients although was 6 times more expensive resulted in 6–8 times greater QALYs (Quality Adjusted Life Years) gained, with cilta-cel having better ICER than ide-cel [27]. The ICER ($123,618) reported by Kapinos et al. was lower than that reported ($253,000) by the Institute for Clinical and Economic Review [28]. The difference could have arisen because Kapinos et al. calculated adverse effects differently and adjusted separately for cytokine release syndrome (CRS) and neurotoxicity. The study did not include indirect costs. The ICER calculated by Kapinos et al. is in comparison to Belantamab as opposed to comparison with a market basket in the study by Institute for Clinical and Economic Review. In Fig. 1 we summarize the Quality-Adjusted Life Years (QALY) Gained for Various Treatments in Multiple Myeloma.

## Challenges and Considerations of Cost Effectiveness of CAR-T Therapy

Overall, the cost-effectiveness of CAR-T therapy in RRMM patients varied based on the drug used (ide-cel vs cilta-cel), PFS data, geographical area, whether indirect costs are considered, comparator used, and methodologic factors like adjusting for adverse effects and sensitivity analysis used. Cost-effectiveness varied based on long term survival with CAR-T therapy. If patients are treatment free during remission this could substantially decrease treatment costs and hence improve cost-effectiveness. Data for cost-effectiveness for CAR-T was based on initial single arm studies, data from randomized control trials (RCTs) and long term follow-up is lacking. Yamamoto et al. suggested that CAR-T may be cost-effective if data from KarMMa-2 or CARTITUDE-2 trial was used as this may result in longer PFS and treatment free intervals given patients with fewer lines of prior treatment in this trial. Studies have suggested that cilta-cel is more cost-effective than ide-cel but it should also be noted that the KarMMa trial for ide-cel although mostly similar population to the CARTITUDE-1 trial for cilta-cel, had more patients with extramedullary disease and high-risk cytogenetics which may be markers of severe disease. Unlike for other hematologic malignancies, CAR-T therapy is not considered curative for MM and virtually all patients progress which affects the cost-effectiveness [29]. Further research in CAR-T therapies addressing antigen escape, trogocytosis, depletion of CAR-T cells, and immune suppression by tumor microenvironment which are implicated as challenges to CAR-T therapy in MM patients can improve outcomes with CAR-T therapy and hence its cost-effectiveness [8, 30–33]. Future studies will be needed to see if the heightened costs of treatment with these novel therapies have an impact on minimizing productivity losses and other indirect costs. Most studies also do not account for indirect costs like transportation, delay for product delivery etc. which can be considered by future studies. In Table 2 we compare the administration time between commonly used regimens and T-cell Engagers in Multiple Myeloma. With the high costs of CAR-T and other newer therapies, sustainability of healthcare systems as in the ability of healthcare systems in delivering expensive treatments to a predominantly older population which at least in the US is majorly publicly insured is an important factor to consider. The increasing financial burden on public insurance programs could strain the ability to provide widespread access to these advanced therapies. Policy changes at federal and state levels are needed to negotiate with drug manufacturers and insurers are needed to bring down costs. Other approaches to include is optimal dosing – moving from once weekly to twice weekly or monthly with equal efficacy outcomes, time limited therapy and avoiding combination therapy unless there is a clearly proven benefit are necessary are some strategies to decrease cost. Further research on cost-effectiveness of different MM regimens will help healthcare providers to find the optimal treatment pathway minimizing costs for both payers and patients.

## Cost-Effectiveness of Bispecific antibodies in Multiple Myeloma Patients

Bispecific antibodies have emerged as promising therapies for triple class refractory multiple myeloma patients based on early phase clinical trials [34]. Bispecific antibodies developed for multiple myeloma currently target B-cell maturation antigen (BCMA), G protein–coupled receptor, class C, group 5, member D (GPRC5D), Fc receptor-like 5 (FcRL5), or CD-38. A recent meta-analysis of prospective clinical trials on 1473 RRMM patients reported an overall pooled ORR of 61% with 28% and 33% of patients achieving complete response in the BCMA and non BCMA bispecific antibody treatment groups respectively. The study also reported any grade neutropenia (48% vs 18%), lymphopenia (38% vs 8%), CRS (64% vs 66%), infections (47% vs 49%), immune effector cell-associated neurotoxicity syndrome(ICANS)(11% vs 2%) as major adverse effects in BCMA and non BCMA groups respectively (35). Currently there are no cost effectiveness studies for bispecific antibodies in multiple myeloma patients likely due to lack of long-term clinical data. The improved efficacy shown in clinical trials may translate into cost-effectiveness, but this should be weighed against the high costs of the drug therapy along with costs involved in potential adverse effect management. Although bispecific antibody therapy is less toxic compared to CAR-T therapy including lower high grade CRS events, it is an ongoing therapy unlike CAR-T which is one time session. Multiple sessions may account for higher costs compared to CAR-T therapy. Bispecific antibody therapy would not incur additional costs from delay in initiation due to production which is an issue with CAR-T therapy. The drug costs may decrease in the future with expansion in the market, increase in bispecific antibody options, limited duration of therapy and use for patients early in the disease spectrum. Future studies investigating the cost-effectiveness of different bispecific antibodies and possible head-to-head comparison to CAR-T therapy will be needed to understand the best options further.

## Conclusions

In conclusion, in this article we review the cost effectiveness of various treatments including CAR-T and Bispecific antibodies. Effective drugs in earlier LOT are more cost effective over the course of time than in later LOT due to longer PFS. CAR-T used in earlier LOT and combined with a longer PFS are likely to be cost effective depending on the country and the model used. Further research is needed for bispecific antibodies and their cost effectiveness in multiple myeloma.

## Key References

- Yamamoto C, Minakata D, Yokoyama D, et al. Cost-effectiveness of anti-bcma chimeric antigen receptor t cell therapy in relapsed/refractory multiple myeloma. Transplant Cell Ther. 2024;30(1):118.e1-118.e15.
  - This study compared the effectiveness of anti BCMA CART to the LOCOMOTION trial.
- Kapinos KA, Hu E, Trivedi J, Geethakumari PR, Kansagra A. Cost-effectiveness analysis of car t-cell therapies vs antibody drug conjugates for patients with advanced multiple myeloma. Cancer Control.
  - This study compared the QALY of CART when compared to other third line therapies in myeloma
- Robinson D, Orlowski RZ, Stokes M, et al. Economic burden of relapsed or refractory multiple myeloma: Results from an international trial. Eur J Haematol. 2017;99(2):119–132.
  - This study shows the indirect costs for patients with myeloma.

## Data Availability

No datasets were generated or analysed during the current study.
